# Host‐specific and deterministic microbiome assembly in major coleopteran stored‐grain pests

**DOI:** 10.1002/imo2.70109

**Published:** 2026-06-11

**Authors:** Abrar Muhammad, Xiaoqiang Shen, Lichao Chen, Enhui Guo, Huarui Zhang, Xiaoyu Lei, Chao Sun, Jianhua Lv, Yongqi Shao

**Affiliations:** ^1^ Max Planck Partner Group, College of Animal Sciences, Faculty of Agriculture, Life and Environmental Sciences Zhejiang University Hangzhou China; ^2^ Analysis Center of Agrobiology and Environmental Sciences Zhejiang University Hangzhou China; ^3^ School of Food Science and Technology, Engineering Research Center of Grain Storage and Security of Ministry of Education, Grain Storage and Logistics National Engineering Laboratory Henan University of Technology Zhengzhou China

## Abstract

Dual DNA‐ and RNA‐based sequencing profiled the microbiomes of five major coleopteran stored‐grain pests and their wheat flour substrate. The microbiomes differed significantly among hosts and from flour, yet 11 core bacterial and 9 core fungal taxa were shared across hosts. RNA data revealed host‐specific active microbiota. Deterministic processes dominated community assembly, exceeding 90% in *Rhyzopertha dominica* and *Oryzaephilus surinamensis*. Homogeneous selection identified eight conserved bacterial clades. Although microbiomes were strongly host‐specific, they showed no significant correlation with host phylogeny, indicating that environmental filtering and deterministic selection, rather than evolutionary relatedness, are the dominant forces shaping these pest‐associated communities.

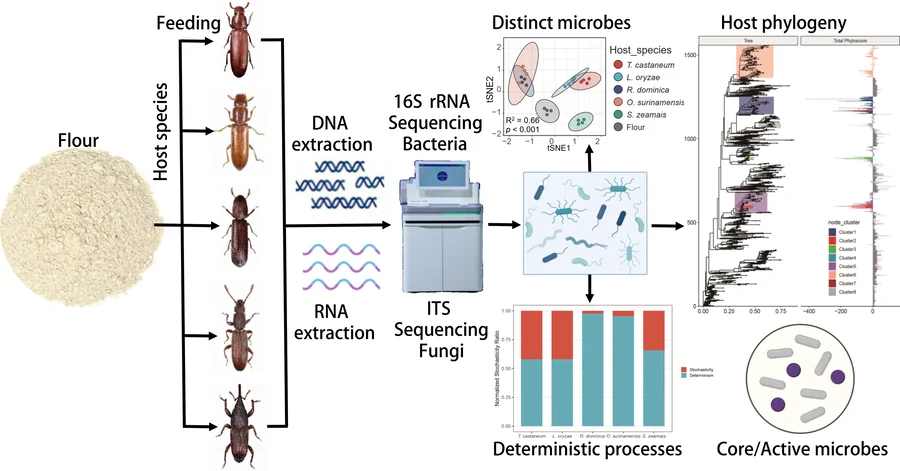

To the Editor,

Stored grain pests pose a significant threat to global food security. High‐value crops suffer not only direct losses during storage, but also quality declines due to contamination and mycotoxin production [1, 2]. As much as 9% of grain is lost after harvest in industrialized countries, and up to 20% in developing regions. These losses can reach tens of millions of tonnes annually and are primarily caused by Coleopteran and Lepidopteran insects, which together account for over 70% of stored‐grain pests [3, 4]. The urgent need for sustainable pest control has increased interest in manipulating insect‐associated microbiota, as symbionts affect nutrition, immunity, and detoxification [5]. However, stored‐grain pests remain understudied, with limited comparative research across species and insufficient consideration of metabolically active taxa. Therefore, we profiled the bacterial and fungal communities of five economically important beetles, including *Tribolium castaneum*, *Latheticus oryzae*, *Rhyzopertha dominica*, *Oryzaephilus surinamensis*, and *Sitophilus zeamais* (Table S1), along with their wheat‐flour substrate. Unlike most DNA‐only surveys, we employed both DNA and RNA amplicon sequencing to capture both total and transcriptionally active microbes. We also assessed community assembly processes and identified conserved taxa and phylogenetically stable clades. Our results indicate that these pests harbor distinct, host‐associated microbiomes primarily shaped by deterministic ecological processes, rather than solely by host phylogeny.

## 1 HOST‑SPECIFIC DIVERSITY AND CORE CONSORTIA

### 1.1 Divergent communities despite a common diet

Bacterial 16S sequencing (DNA‐ and RNA‐based) and fungal internal transcribed spacer (ITS) sequencing (DNA‐based) were used to profile and compare microbial diversity across the pests and the flour substrate (Tables S2–S4). Microbial diversity varied among hosts (Figure 1A,B, Tables S5,S6). Chao1 richness and Shannon diversity analyses revealed that *S. zeamais* harbored the most diverse bacterial community, followed by *T. castaneum*, whereas *R. dominica* and *O. surinamensis* exhibited relatively simple communities (Figure 1A). These patterns were evident in both total and active bacterial datasets, suggesting that host‐specific structuring is not confined to dormant or background taxa but also includes the metabolically active fraction of the microbiome. The common diet of flour supported high microbial diversity but clustered separately from insect‐associated communities (Figure 1C, Table S7). Using t‐distributed Stochastic Neighbor Embedding (t‐SNE), we identified distinct clusters for each beetle species, with flour samples forming a separate cluster (Figure 1C). Fungal patterns were more restricted, as DNA‐based ITS data identified fungal communities in *L. oryzae, R. dominica, S. zeamais*, and flour, but not in *T. castaneum* or *O. surinamensis*. Additionally, fungal RNA was not detected in any of the five pest species. Furthermore, host‐specific taxonomic patterns were more pronounced in RNA data (Figures S1–S3), indicating that active microbiota better reflect true diversity and host interactions than total DNA communities [6, 7]. These patterns indicate strong host filtering despite uniform rearing conditions and suggest that diet alone does not homogenize microbiota.

**Figure 1:**
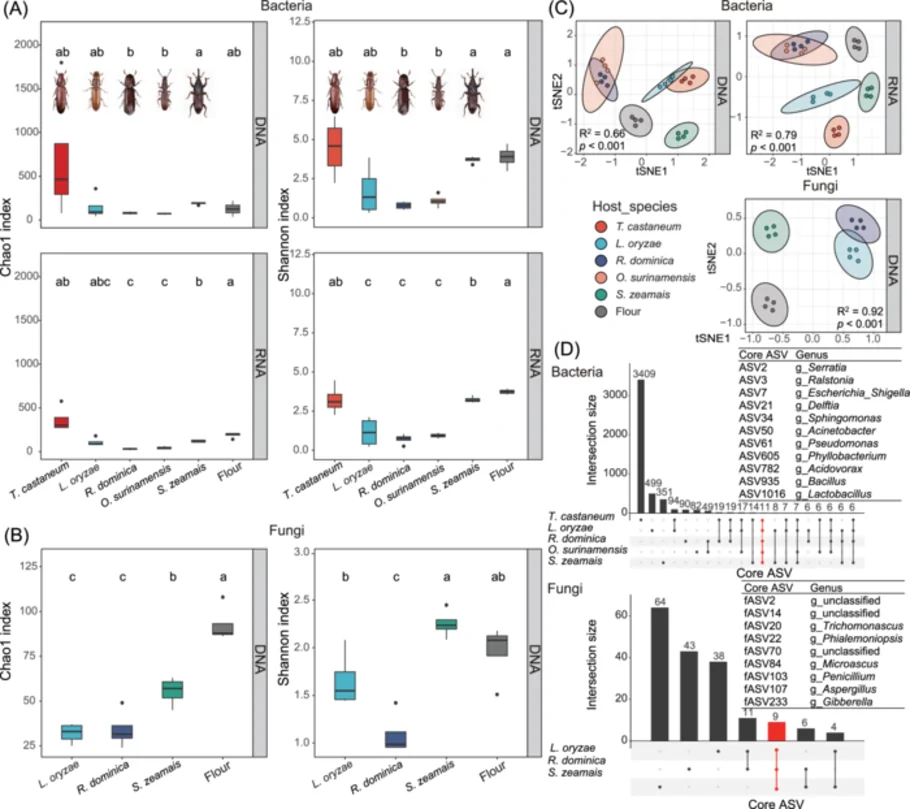
Host‐specific diversity, community structure, and core microbiota of stored‐grain pests and flour substrate. The alpha diversity of bacterial (A) and fungal communities (B) in the DNA‐ and RNA‐based datasets is shown as Chao1 richness and Shannon diversity. Different letters indicate statistical differences among groups based on pairwise *t*‐tests, with *p*‐values adjusted using the Benjamini–Hochberg method. (C) t‐SNE ordination of bacterial communities in DNA‐ and RNA‐based datasets, as well as fungal communities in the DNA‐based dataset. Group separation was evaluated by PERMANOVA based on Bray–Curtis dissimilarities; the reported *R* ^2^ and *p*‐values were derived from this analysis. Full PERMANOVA statistics, including model degrees of freedom, *F*‐values, *R* ^2^, permutation‐based *p* values, and permutation settings, are provided in Table S7. (D) UpSet plots illustrating unique and shared bacterial and fungal ASVs across hosts, with core ASVs shared by all compared hosts listed on the right. ASVs, amplicon sequence variants; t‐SNE, t‐distributed stochastic neighbor embedding.

### 1.2 A conserved core microbiota coexists with host‐specific taxa

Although most amplicon sequence variants (ASVs) were host‐specific, we identified 11 bacterial ASVs present in all pests (Figure 1D). These included *Bacillus*, *Sphingomonas, Phyllobacterium*, *Ralstonia*, *Acidovorax*, *Delftia*, *Acinetobacter*, *Serratia*, *Escherichia‐Shigella*, *Lactobacillus*, and *Pseudomonas*. Their ubiquity across hosts suggests adaptation to both insect and substrate niches, making them promising targets for community‐level interventions. Additionally, nine core fungal ASVs, including *Gibberella*, *Penicillium*, and *Trichomonascus*, among others, were shared across *L. oryzae, R. dominica*, and *S. zeamais* (Figure 1D). However, the inference of fungal roles was limited. ITS sequences were undetectable in *T. castaneum* and *O. surinamensis*, and no fungal RNA was recovered from any species, indicating extremely low fungal biomass or host‐mediated suppression. The absence of fungal amplification likely reflects both biological scarcity and technical biases, such as primer mismatch and extraction inefficiency. Therefore, future work should verify fungal presence using different primers, increased sequencing depth, and qPCR to differentiate between methodological and biological explanations. For stored‐product protection, the core microbiome may facilitate scalable substrate or community‐level interventions, with several taxa demonstrating recognized ecological and functional significance [8, 9, 10].

## 2 ECOLOGICAL ASSEMBLY AND THE INFLUENCE OF HOST PHYLOGENY

### 2.1 Deterministic processes dominate assembly

To disentangle the ecological drivers of microbiome assembly, we applied the normalized stochasticity ratio (NST) (Figure 2A). *R. dominica* and *O. surinamensis* exhibited strongly deterministic assembly (over 90%), whereas *T. castaneum*, *L. oryzae*, and *S. zeamais* displayed a mixture of deterministic (60%–70%) and stochastic (30%–40%) contributions. A dissimilarity‐overlap analysis further confirmed non‐neutral dynamics (Fraction of negative slope (Fns) = 0.02, *p* < 0.05). The high determinism observed in *R. dominica* and *O. surinamensis* coincided with the dominance of vertically transmitted endosymbionts (*Wolbachia*) (Figure S3A), suggesting that strong host‐mediated selection (or low within‐species variability) drives their microbial composition. The tightly regulated microbial environment in *R. dominica* and *O. surinamensis* indicates that these species host finely adapted microbes capable of successful colonization and persistence. This likely reflects long‐term co‐adaptation between the host and microbe, which is critical for their ecological success [11, 12]. In contrast, the mixed assembly mode in *T. castaneum*, *L. oryzae*, and *S. zeamais* suggests that random events, such as microbial dispersal and environmental fluctuations, play a meaningful role in increasing microbial community variability [11, 12, 13].

**Figure 2:**
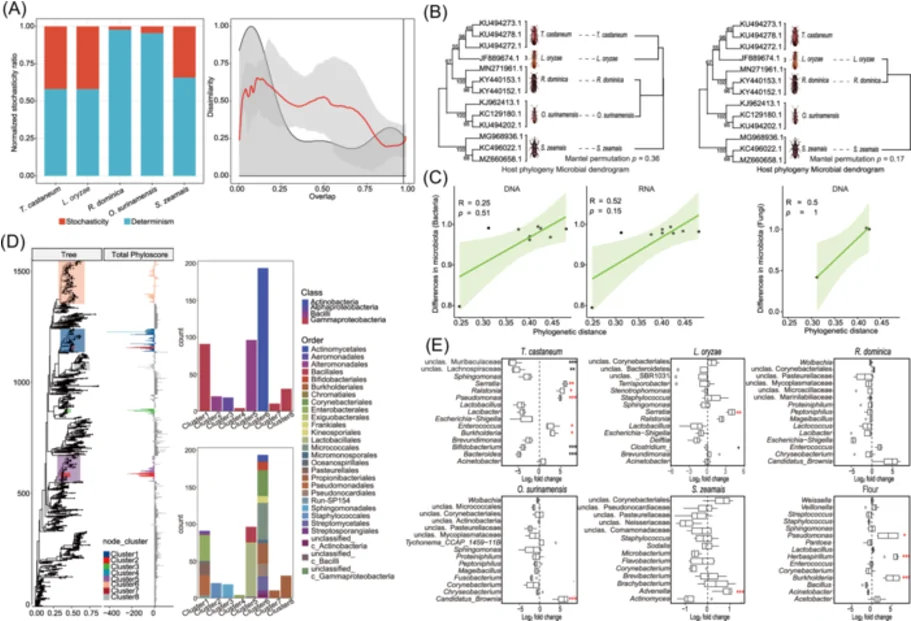
Ecological assembly, phylogenetic relationships, conserved clades, and active bacterial taxa in stored‐grain pest microbiomes. (A) Microbiome assembly processes across the five pest species. Left: the normalized stochasticity ratio indicates the relative contributions of deterministic and stochastic processes. Right: the dissimilarity‐overlap curve compares the observed relationship (red) with the null expectation (black), with shaded areas representing confidence intervals. (B) Comparison of host phylogeny with microbial dendrograms for bacterial and fungal communities. COI sequences were analyzed in MEGA X v10.2.4. The host tree was reconstructed using the Neighbor‐Joining method based on Maximum Composite Likelihood distances, and branch support was evaluated with 1000 bootstrap replicates. The resulting COI‐based distance matrix was used for Mantel permutation tests against host‐level microbial community dissimilarity matrices. No significant correlation was detected for bacteria (five host species, 10 pairwise distances; Mantel *r* = 0.005, *p* = 0.36) or fungi (three host species, three pairwise distances; Mantel *r* = 0.987, *p* = 0.17). (C) Correlations between host phylogenetic distance and microbiota dissimilarity for bacterial DNA, bacterial RNA, and fungal DNA datasets. (D) Homogeneous selection analysis reveals eight conserved bacterial clades, along with their phylogenetic distribution, total phyloScores, and taxonomic composition. (E) Differentially enriched active bacterial genera identified from RNA versus DNA comparisons across each host species and flour substrate. *p*‐values were calculated using the DA.erq2 function in the R package DAtest, which performs a paired differential abundance analysis based on the edgeR quasi‐likelihood model. Significance codes were displayed as: *p < 0.05, **p < 0.01, and ***p < 0.001. Red asterisks indicate genera that were significantly enriched in the RNA‐derived 16S rRNA amplicon dataset relative to the DNA‐based dataset. COI, Cytochrome Oxidase I.

### 2.2 Limited phylogenetic signal

Because the five pest species belong to multiple coleopteran lineages, we also examined whether the microbiome structure aligns with the evolutionary relatedness of the hosts. A host phylogeny was constructed using mitochondrial cytochrome oxidase I (COI) sequences and compared it with Bray–Curtis dendrograms of bacterial communities (Figure 2B). The dendrograms appeared visually congruent, with closely related hosts clustering together; however, Mantel tests revealed no significant correlation between host phylogenetic distances and microbiome dissimilarity (Figure 2C). RNA‐based profiles yielded higher, though still non‐significant, correlation coefficients than DNA profiles. These findings suggest that shared environment and diet can obscure phylogenetic signals and that studying only five host species may be insufficient to detect subtle evolutionary effects. Future studies should incorporate additional taxa and ecological variables, such as digestive physiology and behavior, to clarify the relative roles of host evolution and environmental convergence in shaping insect‐associated microbiota.

## 3 CONSERVED CLADES AND METABOLICALLY ACTIVE MICROBES

### 3.1 Phylogenetic conservation of core lineages

Using a homogeneous selection (HoS) framework, we identified eight bacterial clades with low phylogenetic turnover across hosts (Figure 2D). These clades were enriched in *Actinobacteria*, *Alphaproteobacteria*, and *Bacilli*, highlighting their evolutionary stability under storage conditions. Their persistence suggests that they are not incidental background taxa but evolutionarily and ecologically stable components of the stored‐grain pest microbiome [12, 13]. Importantly, several of these conserved bacterial species overlapped with the core ASVs, suggesting that core taxa are not random but rather belong to evolutionarily conserved lineages that may confer important functions to their beetle hosts [14, 15]. Although conserved fungal ASVs were detected, over 80% of ITS reads in *L. oryzae* and *R. dominica* remained unclassified (Figure S3B), underscoring the need for improved fungal databases and targeted culturing.

### 3.2 Transcriptionally active communities

RNA sequencing enabled us to differentiate between metabolically active and total microbes. Across various hosts, several genera were significantly enriched in RNA profiles compared to DNA, indicating high transcriptional activity (Figure 2E, Table S8). In *T. castaneum*, the genera *Serratia*, *Ralstonia*, *Pseudomonas*, *Enterococcus*, and *Burkholderia* displayed RNA enrichment. *L. oryzae* showed an enrichment of *Serratia*; *O. surinamensis* exhibited increased transcription of *Candidatus Brownia*; and *S. zeamais* showed enrichment of *Advenella*. In flour, active taxa included *Pseudomonas*, *Burkholderia*, and *Herbaspirillum*. Many RNA‐enriched taxa overlapped with HoS clades, reinforcing the notion that conserved lineages are not only metabolically active but also functionally important. Given their versatile metabolic versatility, these genera may play a critical role in nutrient acquisition, detoxification, and the production of antimicrobial compounds [8, 9, 10, 16]. Overall, the convergence of core membership, homogeneous selection, and RNA enrichment strengthens the argument that these microbiomes contain a stable, active bacterial consortium shaped by strong host‐associated ecological filtering.

Our integrated DNA/RNA sequencing of five major coleopteran stored‐grain pests and their flour substrate reveals that host identity significantly shapes microbiota, even with a shared diet. A small core of bacterial taxa is conserved across the pests, and deterministic ecological processes reflecting host filtering and endosymbiont dominance drive community assembly. Phylogenetically stable clades overlap with metabolically active taxa, highlighting their potential functional importance. These findings identify promising targets for microbiome‐based pest management. Future research must verify the functional roles of core microbes, disentangle technical artifacts from biological scarcity (especially for fungi), and validate laboratory observations under realistic storage conditions. Only through such rigorous validation can microbiome manipulation become a practical tool for protecting grain supplies from pest damage.

## AUTHOR CONTRIBUTIONS

**Abrar Muhammad:** Conceptualization; methodology; writing—review and editing. **Xiaoqiang Shen:** Conceptualization; methodology; software; writing—original draft. **Lichao Chen:** Methodology; validation. **Enhui Guo:** Investigation. **Huarui Zhang:** Investigation. **Xiaoyu Lei:** Methodology. **Chao Sun:** Resources. **Jianhua Lv:** Resources. **Yongqi Shao:** Conceptualization; funding acquisition; project administration; resources; writing—review & editing.

## CONFLICT OF INTEREST STATEMENT

The authors declare no conflicts of interest.

## AI STATEMENT

ChatGPT (GPT‐5) was used to improve manuscript clarity. All AI‐assisted text was checked and verified by the authors, who confirm that the final content is correct.

## ETHICS STATEMENT

No animals or humans were involved in this study.

## Supporting information


**Figure S1**: Relative abundance of bacterial communities at the phylum level in the five CSGPs and wheat flour.
**Figure S2:** Relative abundance of fungal communities at the phylum level in *L. oryzae*, *R. dominica*, *S. zeamais*, and wheat flour.
**Figure S3:** Relative abundance of microbial communities at the genus level.

**Table S1:** Taxonomic classification and feeding behaviour of the five major Coleopteran stored grain pests.
**Table S2:** Raw sequence information about bacteria and fungi.
**Table S3:** Summary of denoised bacterial sequence information.
**Table S4:** Summary of denoised fungal sequence information.
**Table S5:** Pairwise *t*‐test results for alpha‐diversity comparisons of bacterial communities are shown in Figure 1A.
**Table S6:** Pairwise t‐test results for alpha‐diversity comparisons of fungal communities are shown in Figure 1B.
**Table S7:** PERMANOVA summary statistics for host‐species effects on microbial community structure in Figure 1C.
**Table S8:** Differentially enriched bacterial genera between RNA‐ and DNA‐derived datasets within each host species and flour.

## Data Availability

The data that support the findings of this study are available from the corresponding author upon reasonable request. The raw sequence data reported in this paper have been deposited in the Genome Sequence Archive in the National Genomics Data Center, China National Center for Bioinformation (GSA: CRA029998), which is publicly accessible at https://ngdc.cncb.ac.cn/gsa/browse/CRA029998. Scripts employed in the computational analyses are available at https://github.com/jxshen233/Insect_analysis. Supplementary materials (methods, figures, tables, graphical abstract, slides, videos, Chinese translated version, and updated materials) may be found in the online DOI or iMetaOmics Science http://www.imeta.science/imetaomics.
