# Supplementary material for: Host‐specific and deterministic microbiome assembly in major coleopteran stored‐grain pests
Source: IMetaOmics. 2026 Jun 11;3(3):e70109. doi: 10.1002/imo2.70109 (PMC13674441; doi:10.1002/imo2.70109)
Supplement: Supplementary file 1 [file IMO2-3-e70109-s001.docx]

Supplementary materials to

**Host-specific and deterministic microbiome assembly in major coleopteran stored-grain pests**

**Running title:** Microbiome assembly in stored grain beetles

Abrar Muhammad^1#^, Xiaoqiang Shen^1#^, Lichao Chen^1^, Enhui Guo^1^, Huarui Zhang^1^, Xiaoyu Lei^1^, Chao Sun^2^, Jianhua Lu^3^, Yongqi Shao^1*^

^1^Max Planck Partner Group, College of Animal Sciences, Faculty of Agriculture, Life and Environmental Sciences, Zhejiang University, Hangzhou 310000, China

^2^Analysis Center of Agrobiology and Environmental Sciences, Zhejiang University, Hangzhou 310000, China

^3^School of Food Science and Technology, Engineering Research Center of Grain Storage and Security of Ministry of Education, Grain Storage and Logistics National Engineering Laboratory, Henan University of Technology, Zhengzhou 450000, China

^#^These authors contributed equally: Abrar Muhammad, Xiaoqiang Shen

^*^Correspondence: yshao@zju.edu.cn (Yongqi Shao)

**METHODS**

**Insect rearing and sample collection**

The five CSGPs used in this study (Table S1) were selected based on their global distribution and economic importance in grain storage systems. *T. castaneum* was kindly provided by Henan University of Technology, China, while the other pest species were obtained from the Qu Shou Insect Botanical Garden company. All species were reared separately under standardized laboratory conditions (30 ± 1°C, 65% relative humidity, and a 12:12 h light: dark photoperiod) to minimize environmental variation in microbiota composition. Wheat flour served as the sole diet and oviposition substrate. Eggs were collected and reared under identical conditions until adult emergence. For each host species, four fully independent biological replicates were established, and each replicate consisted of a pool of five adult individuals. The pooled biological replicate, rather than the individual insect, was used as the statistical unit in all downstream analyses. Newly emerged adults were maintained on fresh flour for 30 days prior to sampling. Adult insects were randomly selected, surface-sterilized with 75% ethanol for 1 min, rinsed three times with sterile deionized water, and homogenized under aseptic conditions using a Precellys-24 homogenizer. Each pooled homogenate was processed as one independent biological sample for the integrated DNA/RNA analysis. Homogenates were immediately processed for nucleic acid extraction.

**Nucleic acid extraction, library preparation, and sequencing**

Genomic DNA and total RNA were extracted from adult insects and wheat flour to capture host-associated and substrate-derived microbiota using the MasterPure^TM^ Complete DNA and RNA Purification Kit (Epicentre, Madison, USA) following the manufacturer’s protocol. Template-free extraction controls were included in each batch to monitor contamination. Nucleic acid quality was assessed using spectrophotometry and gel electrophoresis.

For bacterial community analysis, the V3−V4 hypervariable region of the 16S rRNA gene was amplified using specific primers 338F/806R (ACTCCTACGGGAGGCAGCAG/GGACTACHVGGGTWTCTAAT), while fungal communities were profiled by amplifying the ITS region using primers ITS1/ITS2 (CTTGGTCATTTAGAGGAAGTAA/GCTGCGTTCTTCATCGATGC). PCR reactions were conducted using the following program: 3 min of denaturation at 95 °C, 30 cycles in the next step (30 s at 95 °C, 30 s for annealing at 55 °C (for Bacteria) and 53 °C (for fungi), and 45 s for elongation at 72 °C), and a final extension at 72 °C for 10 min. Amplicon libraries were sequenced on an Illumina MiSeq platform (PE300; Majorbio, Shanghai, China). Negative controls were processed in parallel to detect potential reagent contamination [1]. Briefly, negative extraction controls were processed in parallel using the same DNA extraction workflow as the biological samples. Following PCR amplification with both 16S and ITS primer sets, no visible amplification bands were observed in the negative controls. During the subsequent quality-control step performed by the sequencing provider, these negative-control products did not meet the minimum requirements for library construction due to insufficient amplicon yield; therefore, no negative-control libraries were sequenced. Paired-end reads were processed in LotuS2 v2.25 using the standard pipeline with default settings unless otherwise noted, including quality filtering, ITSx extraction for fungal sequences, denoising with DADA2, chimera removal, and LULU v0.1.0 curation, to generate high-confidence amplicon sequence variants (ASVs) [2]. To minimize biases caused by unequal sequencing depth, all samples were rarefied to 20,847 reads per sample, corresponding to the minimum sequencing depth among all samples after quality filtering. Taxonomic assignment was performed using the SILVA v138.1 (bacteria) and UNITE v9.0 (fungi) databases with a naïve Bayes classifier, using default settings unless otherwise specified [3−5]. Only bacterial and fungal ASVs were retained for downstream analyses. Sequence data are available in the Genome Sequence Archive, China National Center for Bioinformation (GSA: CRA029998).

**Statistical analyses**

All analyses were conducted in R v4.3.2. Alpha diversity (Chao1 and Shannon indices) and beta diversity (Bray-Curtis dissimilarity) were calculated using standard workflows. For alpha-diversity comparisons, pairwise t-tests were used to compare Chao1 richness and Shannon diversity among groups, and *p*-values were adjusted using the Benjamini–Hochberg method to control for multiple comparisons (Tables S5,S6). Community structure was visualized using t-distributed stochastic neighbor embedding (t-SNE), and differences among host species were assessed by PERMANOVA based on Bray-Curtis dissimilarities using the adonis2 function in vegan v2.6.4 with 999 unrestricted permutations (Table S7). Host phylogeny was reconstructed using mitochondrial COI gene sequences. COI sequences were analyzed in MEGA X v10.2.4. The host tree was reconstructed using the Neighbor-Joining method based on Maximum Composite Likelihood distances, and branch support was evaluated with 1000 bootstrap replicates. The resulting COI-based distance matrix was used for Mantel tests against microbial community dissimilarity matrices. Microbial community relationships were evaluated using Bray-Curtis-based dendrograms. Spearman correlations between host phylogenetic distance and microbiome dissimilarity were calculated using ggpubr. The relative contribution of deterministic and stochastic processes to microbiome assembly was quantified using the normalized stochasticity ratio (NST; iCAMP v1.5.12). Community-level ecological dynamics were further assessed using dissimilarity-overlap curve (DOC) analysis (DOC v0.9.0). Phylogenetically conserved and ecologically successful microbial clades were identified using phylofactor (v0.0.1). Microbial differential abundance analysis was performed using unrarefied ASV count data with the DA.erq2 function in the R package DAtest (v2.8.0), based on an edgeR quasi-likelihood model. RNA-versus-DNA enrichment was analyzed using paired comparisons with “sample_number” specified as the pairing factor. *p*-values were adjusted using the Benjamini–Hochberg method, and significance was determined based on FDR-adjusted q values (Table S8).

**SUPPLEMENTARY NOTES (SN)**

**SN. 1 Interpretation of limited fungal detection**

Fungal inference in this study is limited by the absence of fungal RNA across all five pest species and by the non-detection of fungal DNA in *Tribolium castaneum* and *Oryzaephilus surinamensis*. This finding was critically evaluated both biologically and technically. Biologically, fungal biomass may be genuinely low in some host matrices, host filtering may suppress fungal persistence, and some gut environments may be unfavorable for fungal colonization or transcriptional activity [6,7]. Technically, low-abundance fungal templates are more susceptible to underrepresentation due to extraction inefficiency, primer bias in ITS amplification, host-matrix interference, and limited effective sequencing depth [6,7]. Successful bacterial profiling does not exclude methodological bias against fungi, because bacterial and fungal targets differ substantially in abundance, template accessibility, and amplification behavior. Accordingly, the present study supports a cautious interpretation that the fungal associations in these pests remain incompletely resolved, and deeper sequencing, alternative fungal primer sets, targeted qPCR, and culture-based approaches will be required to distinguish biological scarcity from technical under-detection. An additional limitation is taxonomic resolution. More than 80% of ITS reads in *Latheticus oryzae* and *Rhyzopertha dominica* remained unclassified, which substantially limits functional interpretation and prevents strong conclusions about immediate fungal biocontrol relevance without targeted follow-up validation.

**SN. 2 Host phylogeny-microbiome relationships: Statistical and ecological interpretation**

Although the host phylogeny and microbial dendrograms exhibited partial visual congruence, Mantel tests did not reveal statistically significant correlations between host phylogenetic distance and microbiome dissimilarity. Consequently, the current data do not support a strong phylogenetic signal in either the bacterial or fungal datasets. This result must be interpreted considering both statistical and ecological constraints. Statistically, only five host species were included, which inherently limits the number of pairwise comparisons and reduces the power of Mantel-based inference. Additionally, within-species variation diminishes the sensitivity to detect subtle evolutionary effects. Ecologically, all species were maintained under standardized laboratory conditions on the same wheat flour substrate. This shared environment may have fostered environmental convergence, potentially diluting any microbiome differences linked to host evolutionary history. Therefore, while the current design allows for inferences about host-specific microbiome structure under common conditions, it cannot fully disentangle host phylogeny from feeding behavior, digestive physiology, or other host-associated ecological filters.

**SN. 3 Interpretation of RNA-based evidence for active microbiota**

The RNA-based dataset was used to identify taxa that exhibited transcriptional enrichment compared to the DNA-based dataset, suggesting they represent the likely active fraction of the microbiome [8−11]. However, RNA/DNA comparisons should not be viewed as definitive measures of metabolic activity. Ribosome content can vary significantly among bacterial taxa; some lineages can sustain relatively high rRNA levels even under slow-growth conditions, and rRNA may persist when growth is diminished. Therefore, RNA enrichment should be interpreted as evidence of relative transcriptional prominence or physiological engagement, rather than as a direct quantitative measure of metabolic rate [8−11]. In this revised manuscript, RNA-based patterns are discussed in terms of putatively active or transcriptionally enriched taxa.

**SN. 4 Limitation of functional inference and microbiome-based control interpretation**

The functional roles attributed to several core bacterial taxa and HoS clades in this study are derived from taxonomic annotations, their recurrence across different hosts, enrichment in the RNA dataset, and existing literature. However, these inferences are purely correlative. No *in vivo* or *in vitro* functional validation was conducted in this study, so the reported taxa should be considered candidate microbial groups with potential ecological significance, rather than functionally confirmed symbionts. Similarly, microbiome-based control strategies such as RNAi-mediated targeting, phage therapy, or competitive exclusion should be viewed as conceptual future directions rather than validated applications stemming directly from this dataset. Further research, including microbial isolation, colonization, and decolonization assays, targeted expression analyses, metabolite-based validation, and experimental perturbation studies, will be essential before these taxa can be proposed as biocontrol targets with mechanistic support.

**REFERENCES**

1. He, Jintao, Xiaogiang Shen, Nan Zhang, Chao Sun, Yongqi Shao. 2022. “Smartphones as an ecological niche of microorganisms: Microbial activities, assembly, and opportunistic pathogens.” *Microbiology Spectrum* 10: e0150822. <https://doi.org/10.1128/spectrum.01508-22>

2. Özkurt, Ezgi, Joachim Fritscher, Nicola Soranzo, Duncan Y. K. Ng, Robert P. Davey, Mohammad Bahram, Falk Hildebrand. 2022. “LotuS2: an ultrafast and highly accurate tool for amplicon sequencing analysis.” *Microbiome* 10: 176. <https://doi.org/10.1186/s40168-022-01365-1>

3. Yilmaz, Pelin, Laura Wegener Parfrey, Pablo Yarza, Jan Gerken, Elmar Pruesse, Christian Quast, Timmy Schweer, Jörg Peplies, Wolfgang Ludwig, Frank Oliver Glöckner. 2014. “The SILVA and “All-species Living Tree Project (LTP)” taxonomic frameworks.” *Nucleic Acids Research* 42: D643-648. <https://doi.org/10.1093/nar/gkt1209>

4. Nilsson, Rolf Henrik, Karl-Henrik Larsson, Andy F S Taylor, Johan Bengtsson-Palme, Thomas S Jeppesen, Dmitry Schigel, Peter Kennedy, et al. 2019. “The UNITE database for molecular identification of fungi: handling dark taxa and parallel taxonomic classifications.” *Nucleic Acids Research* 47: D259-D264. <https://doi.org/10.1093/nar/gky1022>

5. Bolyen, Evan, Jai Ram Rideout, Matthew R. Dillon, Nicholas A. Bokulich, Christian C. Abnet, Gabriel A. Al-Ghalith, Harriet Alexander, et al. 2019. “Reproducible, interactive, scalable and extensible microbiome data science using QIIME 2.” *Nature Biotechnology* 37: 852-857. <https://doi.org/10.1038/s41587-019-0209-9>

6. Lü, Jun, Shan Xu, Can Teng, Rujia Huang, Guiqin Xiong, Qin Cheng. 2025. “Comparative analysis of gut symbionts in *Tribolium castaneum* (Coleoptera: Tenebrionidae) and their dietary substrate, sauce-flavored Daqu.” *Frontiers in Insect Science* 5: 1614310. <https://doi.org/10.3389/finsc.2025.1614310>

7. Malacrinò, Antonino. 2022. “Host species identity shapes the diversity and structure of insect microbiota.” *Molecular Ecology* 31: 723-735. <https://doi.org/10.1111/mec.16285>

8. Zhang, Nan., Jintao He, Xiaoqiang Shen, Chao Sun, Abrar Muhammad, Yongqi Shao. 2021. “Contribution of sample processing to gut microbiome analysis in the model Lepidoptera, silkworm *Bombyx mori*.” *Computational and Structural Biotechnology Journal* 19: 4658-4668. <https://doi.org/10.1016/j.csbj.2021.08.020>

9. Shi, Weibing, Ryan Syrenne, Jian‐Zhong Sun, Joshua S Yuan. 2010. “Molecular approaches to study the insect gut symbiotic microbiota at the ‘omics’ age.” *Insect Science* 17: 199-219. <https://doi.org/10.1111/j.1744-7917.2010.01340.x>

10. Salgar-Chaparro, Silvia J., Laura L. Machuca. 2019. “Complementary DNA/RNA-based profiling: Characterization of corrosive microbial communities and their functional profiles in an oil production facility.” *Frontiers in Microbiology* 10: 2587. <https://doi.org/10.3389/fmicb.2019.02587>

11. Bashiardes, Stavros, Gili Zilberman-Schapira, Eran Elinav. 2016. “Use of metatranscriptomics in microbiome research.” *Bioinformatics and Biology Insights* 10: 19-25. <https://doi.org/10.4137/BBI.S34610>

**Figure S1 Relative abundance of bacterial communities at the phylum level in the five CSGPs and wheat flour.** (Upper panel) DNA-based sequencing data representing the total microbial community composition. (Lower panel) RNA-based sequencing data indicating metabolically active bacterial taxa.

**Figure S2 Relative abundance of fungal communities at the phylum level in *L. oryzae, R. dominica, S. zeamais*, and wheat flour.** Data are based on ITS sequencing (DNA).

**Figure S3 Relative abundance of microbial communities at the genus level.** (A) Bacterial composition based on DNA sequencing data (upper panel) and RNA sequencing data (lower panel). (B) Fungal composition based on DNA sequencing data.
